# PaLS Study: Tobacco, Alcohol and Drugs Usage among Polish University Students in the Context of Stress Caused by the COVID-19 Pandemic

**DOI:** 10.3390/ijerph19031261

**Published:** 2022-01-23

**Authors:** Alicja Monika Jodczyk, Przemysław Seweryn Kasiak, Natalia Adamczyk, Joanna Gębarowska, Zuzanna Sikora, Grzegorz Gruba, Artur Mamcarz, Daniel Śliż

**Affiliations:** 1 Students’ Scientific Group of Lifestyle Medicine, 3rd Department of Internal Medicine and Cardiology, Medical University of Warsaw, 04-749 Warsaw, Poland; alajodczyk4@gmail.com (A.M.J.); adamczyk.natalia1997@gmail.com (N.A.); gebarowska.joanna@gmail.com (J.G.); zznnsikora@gmail.com (Z.S.); grzegorz@gruba.pl (G.G.); daniel.sliz@wum.edu.pl (D.Ś.); 2 Polish Society of Lifestyle Medicine, 00-388 Warsaw, Poland; artur.mamcarz@wum.edu.pl; 3 3rd Department of Internal Medicine and Cardiology, Medical University of Warsaw, 04-749 Warsaw, Poland; 4 School of Public Health, Postgraduate Medical Education Center, 01-813 Warsaw, Poland

**Keywords:** tobacco, cigarettes, alcohol, COVID-19, students, public health

## Abstract

The COVID-19 pandemic and imposed restrictions were strong stress factors for young people, especially students. Increased alcohol consumption, smoking cigarettes, usage of heated tobacco products, and other stimulants are common methods of coping with anxiety. However, they can have serious negative health effects. A survey consisting of 12 questions related to mental health and psychoactive substance taking habits was distributed among Polish students between 22 February 2021 and 3 April 2021. A total of 1323 participants met all inclusion criteria (*n*_females_ = 1021, *n*_males_ = 297, *n*_other gender_ = 5). The mean age was 22 years old (±4.17); 47.62% were medical university students. A total of 71.92% reported negative impact, 8.25% did not notice changes, and 12.58% declared a positive pandemic impact on their mental health. A total of 12.58% declared an increase, 70.22% did not see any differences, and 17.20% reported a decrease in their psychoactive substance usage tendency due to the pandemic. Worse perceived psychologic well-being was correlated with a higher tendency to use tobacco (*p* < 0.001) and alcohol (*p* < 0.001), and not with marijuana and products containing tetrahydrocannabinol (*p* = 0.136), and hard drugs (*p* = 0.799). The majority of participants declared a negative pandemic impact on mental health and did not report significant changes in psychoactive substance taking habits. Medical personnel should be aware of the current situation and apply for proper prevention and treatment programs.

## 1. Introduction

The COVID-19 disease, caused by severe acute respiratory syndrome coronavirus 2 (SARS-CoV-2), is a novel problem in public health and was declared as a global pandemic by the World Health Organization (WHO) on 11 March 2020. The scale of the pandemic has resulted in a worldwide concern, not only for health problems but also social and economic impacts [1]. The Polish Government announced the epidemic in Poland on 20 March 2020 [2]. The outbreak has affected the entire globe and influenced all aspects of everyday life. Numerous restrictions, which aimed to limit the spread of the virus (such as: staying at home, quarantine periods, closures of basic facilities and services) were introduced. Although these actions were necessary [3,4], it is already known that long quarantine periods entail negative consequences for mental health. The review of the epidemiology of mental health problems in COVID-19 revealed an increase in depression, anxiety and mood disorders, stress, panic, anger, impulsivity and deterioration of the quality of sleep in different populations [5]. Additionally, the research included in the analysis showed that individuals affected by COVID-19 may have a higher burden of mental problems than non-affected ones [5]. Uncertainty, fear for oneself and relatives, difficulties in adapting to the new situation, and dealing with many unpleasant emotions and situations lead to difficult psychological consequences. Some people may manage social isolation and any pandemic-related psychological distress by commencing or increasing adverse health behaviors, such as smoking or alcohol use [1]. It has been proved that alcohol and other psychoactive substances inhibit the nervous system, and thus are commonly used by people seeking relief from difficult situations and depression [6]. Students cope with stress in a variety of ways including adaptive strategies, e.g., alcohol consumption, smoking, and use of psychoactive substances [7]. Overusing alcohol is an alarming problem among young people, including students [8,9]. In comparison to the general adult population, their alcohol intake is significantly higher [10]. It is estimated that about one in three people who abuse alcohol in their youth become addicted [7].

Tobacco smoking is another concerning problem [11]. The negative effects of smoking on overall health have been well documented. Smokers have a higher risk of heart diseases, lung cancer, respiratory infections and asthma. [12,13]. They live, on average, ten years shorter than non-smokers [14]. E-cigarettes and heated tobacco products (HTP) are a form of nicotine delivery intended to provide an alternative to traditional cigarettes. Tobacco companies claim that HTP are less harmful than traditional cigarettes but their potential impact on human health has not been fully investigated yet [15,16]. The prevalence of alternative smoking has been increasing, especially in highly developed countries [17]. Some research suggests that e-cigarettes may have a higher addictive potential than traditional ones, as they are used in times and places where smoking is prohibited [16]. Young people often think that those products are less harmful and addictive than traditional ones. The number of people who start smoking tobacco from alternative forms constantly rises [18]. Even though e-cigarettes are promoted to help quit smoking, the findings on that point are mixed. Data show that 40% of US e-cigarette users aged 18–24 years in 2015 had never been regular cigarette smokers [19].

The report on drug addiction conducted by the National Office for Counteracting Drug Addiction in 2019 revealed that the consumption of alcoholic beverages in Poland is much more widespread than drug use [20]. Here, “drugs” mean all addictive substances, which are used to experience pleasure, a certain mood and extreme sensation (such as marijuana, amphetamine, heroin, methamphetamine, cocaine, etc.) and are illegal in Poland. Moreover, the scale of narcotics use in Poland in comparison with other countries of the European Union is not big. Marijuana is the most commonly used drug. Data show that Polish citizens seek treatment from the abuse of three main groups of substances: marijuana and hashish, stimulants and opioids.

The COVID-19 pandemic has changed entirely the lifestyle of Polish University Students due to the transition of lectures into online learning and limitations in their interactions with other people. Our study aimed to evaluate the impact of the COVID-19 pandemic on drinking alcohol, smoking traditional and alternative cigarettes, usage of marijuana, products containing tetrahydrocannabinol (THC) and hard drugs (such as heroin, cocaine, methamphetamine and amphetamine) among university students in Poland. Overall, we hypothesized that the pandemic situation would be positively correlated with tobacco, alcohol, marijuana, THC products and hard drugs usage, negatively correlated with mental health and that individuals with poorer mental health would have a higher alcohol, tobacco, marijuana and hard drugs intake.

## 2. Materials and Methods

### 2.1. Study Design

The sample of 1646 students fulfilled the self-administered questionnaire, which was shared via e-mails and social media channels (e.g., the Polish Lifestyle Medicine newsletter, students’ university groups, or Facebook). After the two-stage data cleaning process, 1323 of them qualified for the analysis. Data were collected between 22 February 2021 and 3 April 2021 during half-term time and the beginning of the second academic semester (nearly a year after the pandemic outbreak in Poland). The participant had to have a status of a bachelor’s or Master’s degree student or equivalent. Although there were no age limitations PhD students and postgraduate students were not included. The terms of participation and the information about the anonymity of the study were stated directly at the beginning of the survey. Fulfilling the form and submitting it was taken as consent to participate in the study. Our study did not require Institutional Review Board approval and a proper judgment was obtained. It was a part of the PaLS—“Pandemic against LifeStyle” project—which aimed to evaluate the impact of the lockdown on the lifestyle (nutrition, physical activity, mental health, sleeping behaviour, and psychoactive substances usage) among university students in Poland. Articles about sleep deprivation, mental health consequences, physical activity decrease, and changes in dietary habits have been already published [21,22].To analyze physical activity, the Physical Activity Questionnaire—Short Form (IPAQ-SF) was used. Thanks to the scoring protocol of the form, students who did not remember their physical activity habits had to be removed from the analysis [23]. Additionally, unviable answers (e.g., BMI = 5 kg/m^2^ or being physically active for 24 h/day) were excluded. There were no other exclusion criteria.

### 2.2. Construction of the Questionnaire

The questionnaire relating to psychoactive substance usage consisted of three parts. (1) Demographic characteristics such as age, sex, and type of the university: medical university students (MUS)/non-medical university students (NMUS);(2) questions about the frequency of drinking at least one portion of alcohol (never/occasionally/once a month/once a week/3–4 times a week/every day), amount of traditional cigarettes smoked daily (0/<10/10–19/20 and more), and frequency of alternative tobacco products usage such as e-cigarettes, HTPs, IQOS and waterpipes (never/several times per year/less than 20 days; within 30 days/20 days; within 30 days or often/up to an hour every day); (3) The authors’ questions scaled −5/0/+5 about responders’ subjective assessment of the pandemic’s impact on their mental health and tendency to alcohol, tobacco, marijuana, products containing THC and hard drugs intake. In the question about changes in mental health answers from −5 to −1 meant that the pandemic had a negative impact, answer 0 meant no effect, and answers from +1 to +5 meant positive impact (“−5” meant the most negative impact, “+5”—the most positive impact). In questions about changes in psychoactive substance taking habits, answers from −5 to −1 meant that the pandemic resulted in a reduction in current usage or tendency to use, 0 no change, +1 to +5 an increase (“−5” meaning the highest reduction, “+5” the highest increase). The questionnaire form is available in the Supplementary Material.

### 2.3. Data Analysis

All statistical analyses were performed using the statistical software STATISTICA (version 13.3, StatSoft Polska Sp.z. o.o., Kraków, Poland) and SPSS Statistics (version 27.0, IBM, Chicago, IL, USA). Basic statistical calculations were made as follows: normal distribution, mean, and standard deviation (SD). Data about quantity and frequency were expressed as percentages. Based on mean ranks and statistical test scores, the direction of correlation (positive or negative) was assessed. Shapiro–Wilk test was used to assess normal data distribution. To calculate correlations between −5/0/+5 self-opinion question about mental health and −5/0/+5 self-opinion questions about psychoactive substances usage non-parametric Rho–Spearman test was performed. To assess the correlation between answers in −5/0/+5 self-opinion questions about psychoactive substances usage and gender or studying at the medical university U-Mann–Whitney test was performed. Statistical significance borderline was *p* = 0.05.

## 3. Results

A total number of 1646 Polish students participated in the study, 1323 of them met all of the study conditions. Among them 77.17% (*n* = 1021) were females, 22.45% (*n* = 297) were males, 0.38% (*n* = 5) did not specify gender. The mean age of the participants was 22.23 years old (SD = 4.17) and the median was 22 years old. MUS represented 47.62% (*n* = 630) of responders, while 52.38% (*n* = 693) were NMUS.

### 3.1. Frequency and Quantity Questions

Table 1 presents the percentage of particular groups: men, women, MUS, NMUS and a total number of participants smoking traditional cigarettes on a normal day during the previous year (the time of the COVID-19 pandemic). The majority of any of this group declared being a non-smoker.

Table 2 presents the percentage of particular groups of participants: women, men, MUS, NMUS and a total number of participants smoking alternative tobacco forms on a normal day during the previous year (the time of the COVID-19 pandemic).

A total of 12.32 % of responders (16.5 % of male and 11.07% of female) smoked traditional cigarettes regularly, while 28.19% of responders (34.34% of male and 26.54% of female) admitted that they were users of alternative tobacco products (they used them at least occasionally). A total of 7.71% of our responders were dual smokers (62.58% of traditional cigarettes smokers were also alternative smokers).

Table 3 presents the frequency of drinking alcohol by particular groups of participants on a normal day during the previous year (the time of the COVID-19 pandemic): women, men, MUS, NMUS, and the total number of participants.

The total number of 12.02% of responders were non-drinkers. This number was higher for women (12.24%) than for men (10.44%). A total of 58.5% of all responders drank alcohol regularly (once a month or often). This number was higher for men (67.37%) than for women (56.12%).

### 3.2. Self-Opinion Questions

**Figure 1:**
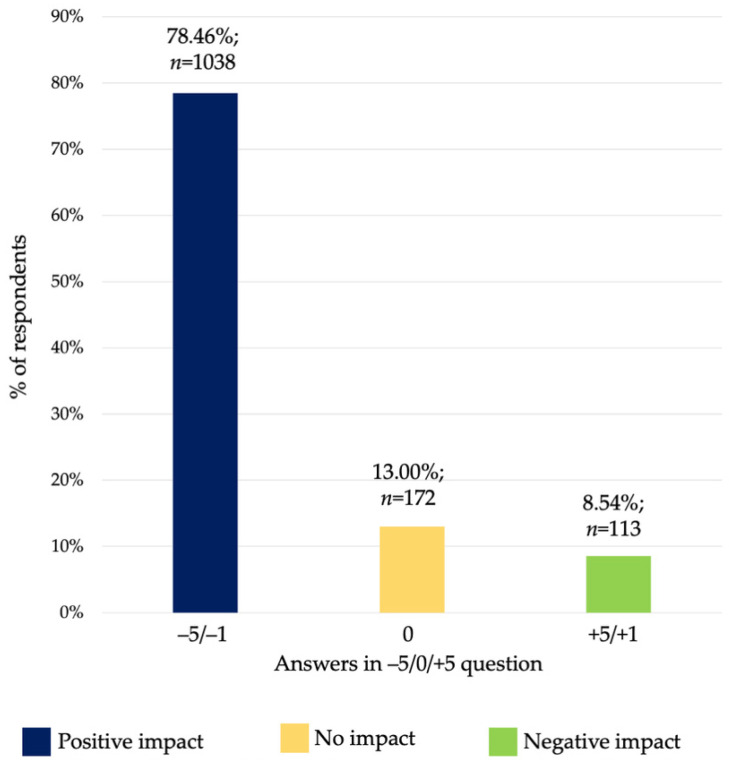
Declared impact of COVID-19 pandemic on mental health. Numbers from “−5” to “−1” meant a reduction in usage and from “+1” to “+5” an increase.

Results of the declared impact of the COVID-19 pandemic on mental health are presented in Figure 1. A total of 78.46% of the responders described the impact of the pandemic on their mental health as negative (answers from “−5” to “−1”), 13% did not notice any change (answer “0”) and 8.54% perceived a positive impact (answers from “+1” to “+5”).

**Figure 2:**
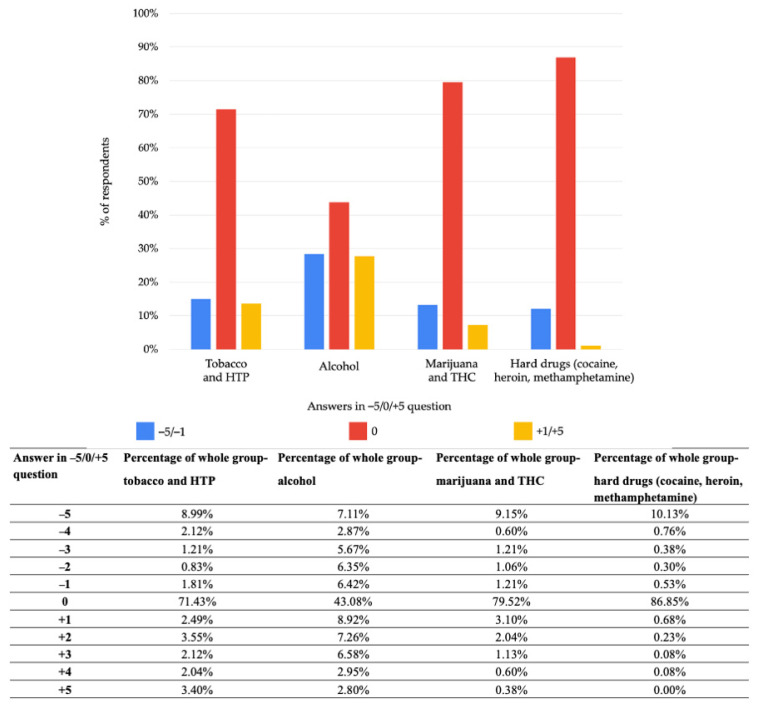
Declared impact of COVID-19 pandemic on alcohol, tobacco, marijuana and THC and hard drugs usage tendency. Answers from “−5” to “−1” meant reduction in usage, answer “0” no change, answers from “+1” to “+5” meant an increase. Abbreviations: HTP, heated tobacco products; THC, products containing tetrahydrocannabinol.

Results of declared impact of COVID-19 pandemic on alcohol, tobacco, marijuana and THC and hard drugs (such as heroin, cocaine, methamphetamine) usage tendency are presented in Figure 2. Most of the responders did not notice any changes during the pandemic (answer “0”). Numbers from “−5” to “−1” meant a reduction in usage and from “+1” to “+5” an increase.

- Alcohol: 28.41% (*n* = 377) increased tendency, 43.08% (*n* = 570) no change, 28.51% (*n* = 377) decreased tendency;
- Tobacco: 13.61% (*n* = 180) increased tendency, 71.43% (*n* = 945) no change, 14.96% (*n* = 198) decreased tendency;
- Marijuana and THC: 7.25% (*n* = 96) increased tendency, 79.52% (*n* = 1052) no change, 13.23% (*n* = 175) decreased tendency;
- Hard drugs: 1.05% (*n* = 14) increased tendency, 86.85% (*n* = 1149) no change, 12.1% (*n* = 160) decreased tendency;
- Average answer: 12.58% increased tendency, 70.22% no change, 17.20% decreased tendency.

The second most frequently chosen answer was “−5”, which meant the highest possible decrease in usage of a specific product. This response was chosen by 7.11% (*n* = 94) of participants in questions about alcohol, 8.99% (*n* = 119) in questions about tobacco, 9.15% (*n* = 121) in questions about THC and marijuana and 10.13% (*n* = 134) in questions about hard drugs.

Poorer self-opinion on a pandemic impact on mental health was correlated with higher self-assessed intake of alcohol and tobacco and was not significantly correlated with higher marijuana and THC and hard drugs usage. Data are presented in Table 4. Statistically significant data are presented in bold.

Gender was not significantly correlated with a higher self-perceived tendency to drink alcohol (*p* = 0.075), smoke cigarettes (*p* = 0.323), usage of marijuana and THC (*p* = 0.683) or hard drugs (*p* = 0.669). Being a MUS was not significantly statistically correlated with a higher self-perceived tendency to drink alcohol (*p* = 0.299), smoke cigarettes (*p* = 0.601), use marijuana and THC (*p* = 0.088), or hard drugs (*p* = 0.079).

## 4. Discussion

Our findings suggest that the majority of our responders did not notice significant changes in alcohol, tobacco, marijuana and THC and hard drugs intake resulting from the pandemic situation. Nearly the same number of students reported a decrease and increase in both drinking alcohol and smoking. Much more participants noted a decrease than an increase in usage of marijuana, and products containing THC and hard drugs. Most of the responders who reported a decrease in alcohol, tobacco, marijuana, and hard drugs intake chose that it had decreased in the highest possible way (answer “−5”). Those findings can be explained by the social customs connected with using drugs, tobacco, and alcohol in Poland. Studies are the time when many students move to different cities, live with roommates, or in dormitories where they often gather and party together. Previous research showed that an increase in the consumption of alcoholic drinks was accompanied by an increase in overall drinking occasions [24,25]. The closure of universities and social gathering sites combined with the onset of online classes could cause a significant decrease in drinking occasions [24].

A decrease in self-perceived psychological well-being was significantly related to self-estimated higher consumption of alcohol and tobacco and was not significantly correlated with self-perceived usage of marijuana and products containing THC, or hard drugs for general study population. Although, for particular subgroups results slightly differ, the major direction of changes has been observed (see Table 4). The negative effect of the COVID-19 pandemic on mental health is commonly known [22,26]. A different study conducted at the beginning of the pandemic has also revealed that those students who had reported depression or anxiety demonstrated a higher increase in alcohol consumption [27]. Long quarantine duration, infection fears, frustration, boredom, inadequate information had impacted mental health and well-being. In those stressful situations, people tended to overuse stimulants [28]. Therefore, it is particularly important to pay attention to people with poor psychological conditions, to prevent them from becoming addicts.

Tobacco users were divided into traditional cigarettes smokers and alternative products containing tobacco (such as e-cigarettes, IQOS, HTPs) users. According to the data provided by the Chief Sanitary Inspectorate from 2019, more than one-fifth of Poles admit to habitual (every day) smoking of traditional cigarettes [29]. This result is lower than in the previous survey, conducted in 2017. The research showed that men were more frequent smokers than women (24% vs. 18%). The percentage of smoking men was increasing almost proportionally with age. In the age group of 20–29, 7% of women and 24% of men admitted to habitual smoking. In the case of electronic cigarettes (e-cigarettes), the same data showed that 1% of all Poles smoke e-cigarettes, and 4% of them declared that they had used them at least once. The most frequently indicated reasons for smoking e-cigarettes were the fashion for smoking them (28%), a belief that e-cigarettes are better for health (17%), saving money (14%) and willingness to quit smoking (13%). Comparing our respondents with the data from the population of age 20–29 we can notice a lower percentage of men (16.5% vs. 24%) and a higher percentage of women (11% vs. 7%) smoking traditional cigarettes. Our study also revealed that a significantly higher percentage of our responders reached for alternative cigarettes (used them at least once) in comparison to the data from 2019 (28% vs. 4%). The commonly observed phenomenon is dual usage (simultaneous use of traditional and alternative cigarettes). More than half of our respondents, who were traditional smokers were also dual smokers. As previously mentioned, alternative products containing tobacco are gaining popularity especially among adolescent and younger populations [17,30]. They are more attractive for young people due to a variety of flavors, especially the sweet fruit variants. Despite they are considered to be less harmful, they can be more addictive than traditional ones and their long-term effect on the human body is still unknown [16]. Numerous studies have reported that current smokers, cigarette users or dual users are more likely to perceive e-cigarettes as safe for health [30,31]. According to the World Health Organization statement, there is no evidence that HTPs are less harmful than conventional cigarettes [32]. The risk of respiratory tract infections in smokers may be increased through various mechanisms. It increases the risk of several types of pneumonia infection and influenza [33]. From the analysis of available studies, it appears that patients with a history of smoking have a higher likelihood of developing more severe symptoms of COVID-19 disease than non-smokers [34]. Moreover, smokers have a higher probability of COVID-19 progression [35]. Electronic cigarettes and other alternative devices are unlikely to be a safer option regarding COVID-19-related risks. Since they use tobacco and produce smoke or vapor, they may cause the infectious lung damage observed with traditional cigarettes [33].

The two reports considering alcohol drinking, first from the State Agency for Solving Alcohol Problems, and second from the Public Opinion Research Center from 2019, showed an increase in alcohol consumption in Poland [36,37]. Between 2010 and 2019, the number of men who regularly drink alcohol decreased, but the number of regularly drinking women increased. A 58.5% of our responders drank alcohol regularly (once a month or often). This number was higher for men (67.37%) than for women (56.12%). In addition, people who reported more frequent intake of alcohol also reported a worse self-perceived pandemic impact on that intake. More than one-fourth of responders reported that they drank more alcohol than before the pandemic. Another study conducted at the beginning of the pandemic revealed that over 28% of its participants had drunk at risk levels [25]. Alcohol consumption is a common problem among Polish students [38,39] and it increases among both males and females [39]. A pre-pandemic study among university students revealed a two-fold increase in the number of Polish male and female students who abused alcohol to blackout levels between 2000 and 2016 [39]. The same study revealed an increase in the number of students drinking alcohol once a month and a decrease in the number of abstainers [39]. Alcohol increases aggression by causing changes within the person organism, e.g.,: it reduces intellectual functioning [40]. High alcohol intake is closely linked to the risk and severity of interpersonal violence, such as intimate partner violence and sexual violence [41].

There were no significant differences between gender and declared self-perceived pandemic impact on the usage of the considered substances. However, a slightly higher percentage of men admitted to smoking traditional cigarettes (16.5% of males and 12.32% of females smoked at least one cigarette a day). A higher proportion of men reached for alternative cigarettes (34.34% of men and 26.54% of women smoked them at least occasionally). This is in line with previous reports from Poland [30], Europe, and the United States [42,43], which revealed that women were less likely to reach for cigarettes. In our study, a higher percentage of women declared abstinence (10.44% men vs. 12.24% women). It is known that women drink lower amounts of alcohol than men and, in consequence, have fewer problems with the law, family, and health. Moreover, their risk of death from over usage of alcohol is also lower [39]. Compared to men, more women are lifetime abstainers, drink less, and are less likely to engage in problem drinking, develop alcohol-related disorders or alcohol withdrawal symptoms [44,45].

There were also no strong differences between being a MUS and the reported self-perceived worst pandemic impact of any of the intake of the considered substance. The lower percentage of MUS were abstinent (9.52% MUS compared with 14.29% NMUS) but a higher percentage of them were non-smokers of traditional cigarettes (90.48% MUS compared with 85.14% NMUS). Nearly the same percentage of those groups admitted to smoking alternative cigarettes at least occasionally (72.53% MUS compared with 71.14% NMUS).

## 5. Conclusions

The presented findings paint a picture of changes in the usage of alcohol, tobacco, marijuana, THC and hard drugs among university students in Poland during the SARS-CoV-2 pandemic. The main findings are: (1) majority of the students did not notice changes in alcohol, tobacco, marijuana and THC and hard-drug taking habits; (2) most of the respondents who reported decreased usage of those substances thought that it decreased in the strongest possible way, (3) the worst pandemic effect on mental health was highly correlated with a higher tendency to drink alcohol and smoke tobacco and not correlated with the usage of marijuana and products containing THC and hard drugs; and (4) alternative tobacco products were more popular among university students than classic cigarettes during the pandemic. Minimizing the usage of psychoactive substances and improving psychological well-being are still essential factors of complex prevention programs in the health care system. For a better assessment of the impact of the COVID-19 pandemic on the psychoactive substance intake of Polish undergraduates, more long-term studies are needed.

## 6. Limitations

Our research was carried out online and there is a probability that the questions were not clear enough for respondents. Additionally, we cannot be sure that they truly fulfilled the inclusion criteria. The study sample was opportunistic, relatively small and may not reflect the situation of the entire population. A significantly higher number of our responders were females, which can be related to the fact that in Poland they were more willing and discussed the COVID-19 pandemic on Facebook and Instagram [46]. Those were two channels, which we used most often to spread our survey. There is a probability that psychoactive substances usage varied during the whole pandemic period and we asked only about the average values. In addition, data before imposed restrictions were not collected since our study was conducted nearly a year after the pandemic outbreak. In our opinion, the risk that the responders would not remember their customs after such a long time was too high. It is hard to determine whether participants started, continued, or gave up usage of particular substances during the pandemic because we did not have a direct question on this point. Our questionnaire also did not include an additional question about the detailed amount of marijuana and products containing THC, or hard drugs usage. The data cleaning process was implemented to reduce the impact of the limitation. Responders whose answers were unviable were excluded from the analysis.

## Figures and Tables

**Table 1:** Number of traditional cigarettes smoked on a normal day during the previous year (the time of the COVID-19 pandemic).

Variable	Total (100%; *n* = 1323)	Men (22.45%; *n* = 297)	Women (77.17%; *n* = 1021)	MUS (47.62%; *n* = 630)	NMUS (52.38%; *n* = 693)
0	87.68%	83.50%	88.93%	90.48%	85.14%
<10	10.28%	13.47%	9.30%	8.06%	12.27%
10–19	1.66%	2.36%	1.47%	1.11%	2.16%
20 and more	0.38%	0.67%	0.29%	0.32%	0.43%

Abbreviations: MUS, medical university Students; NMUS, non-medical university students.

**Table 2:** Amount of alternative tobacco forms smoked during the previous year (the time of the COVID-19 pandemic).

Variable	Total (100%; *n* = 1323)	Men (22.45%; *n* = 297)	Women (77.17%; *n* = 1021)	MUS (47.62%; *n* = 630)	NMUS (52.38%; *n* = 693)
Never	71.81%	65.66%	73.46%	72.54%	71.14%
Several times per year (occasionally)	18.07%	21.21%	17.24%	18.41%	17.75%
Less than 20 days within 30 days	2.12%	3.37%	1.77%	1.75%	2.45%
20 days within 30 days or often	1.51%	1.35%	1.57%	1.59%	1,44%
Up to an hour every day	3.17%	5.05%	2.64%	3.49%	2.89%
More than an hour every day	3.33%	3.37%	3.33%	2.22%	4.33%

Abbreviations: MUS, medical university students; NMUS, non-medical university students.

**Table 3:** Frequency of drinking alcohol during the previous year (the time of the COVID-19 pandemic).

Variable	Total (100%; *n* = 1323)	Men (22.45%; *n* = 297)	Women (77.17%; *n* = 1021)	MUS (47.62%; *n* = 630)	NMUS (52.38%; *n* = 693)
Never (non-drinkers)	12.02%	10.44%	12.24%	9.52%	14.29%
Several times per year (occasionally)	29.48%	22.22%	31.64%	32.22%	26.99%
Once a month	23.20%	25.59%	22.62%	24.60%	21.93%
Once a week	25.85%	29.97%	24.68%	22.86%	28.57%
3–4 times a week	8.39%	9.76%	8.03%	10%	6.93%
Every day	1.06%	2.02%	0.78%	0.79%	1.30%

Abbreviations: MUS, medical university students; NMUS, non-medical university students.

**Table 4:** Correlations between the perceived impact of COVID-19 pandemic on mental health and tendency to drink alcohol, smoke tobacco, usage of marijuana, and products containing THC, and hard drugs.

Correlation between the Perceived Impact of COVID-19 Pandemic on Mental Health (Lower Score in −5/0/+5 Question) on the Tendency to (Higher Score in −5/0/+5 Question):	Rho–Spearman Test Scores				
Variables	Total (100%; *n* = 1323)	Men (22.45%; *n* = 297)	Women (77.17%; *n* = 1021)	MUS (47.62%; *n* = 630)	NMUS (52.38%; *n* = 693)
Smoke tobacco	Rho = −0.128; ***p*** **< 0.001**	Rho = −0.111; *p* = 0.056	Rho = −0.139; ***p*** **< 0.001**	Rho = −0.124; ***p*** **= 0.002**	Rho = −0.128; ***p*** **< 0.001**
Drink alcohol	Rho = −0.073; ***p*** **< 0.001**	Rho = −0.053; *p* = 0.365	Rho = −0.075; ***p*** **= 0.017**	Rho = −0.085; ***p*** **= 0.032**	Rho = −0.067; *p* = 0.079
Usage of marijuana and THC	Rho = −0.041; *p* = 0.136	Rho = −0.132; ***p* = 0.023**	Rho = −0.012; *p* = 0.709	Rho = −0.015; *p* = 0.704	Rho = −0.064; *p* = 0.090
Usage of hard drugs	Rho = −0.007; *p* = 0.799	Rho = −0.098; *p* = 0.093	Rho = −0.017; *p* = 0.586	Rho = −0.047; *p* = 0.239	Rho = −0.023; *p* = 0.537

Abbreviations: THC, products containing tetrahydrocannabinol, MUS, medical university students; NMUS, non-medical university students.

## Data Availability

The data presented in this study are available on request from the corresponding author. The data are not publicly available due to not obtaining consent from respondents for publishing the data.

## Supplementary Materials

The following are available online at https://www.mdpi.com/article/10.3390/ijerph19031261/s1. S1: Questionnaire form.
