# Supplementary material for: PaLS Study: Tobacco, Alcohol and Drugs Usage among Polish University Students in the Context of Stress Caused by the COVID-19 Pandemic
Source: Int J Environ Res Public Health. 2022 Jan 23;19(3):1261. doi: 10.3390/ijerph19031261 (PMC8835269; doi:10.3390/ijerph19031261)
Supplement: Supplementary file 1 [file ijerph-19-01261-s001.zip › ijerph-1492321-supplementary.pdf]

Supplementary material- Questionnaire form

ENGLISH Version

Part 1.- general and demographic questions

1. Gender

_____ (male, female, other, I would rather not to give it)

2. Height

_____ (in cm)

3. Weight

_____ (in kg)

4. Age

_____ (in years)

5. Are you a Medical University student? (Fields which include you into Medical University students: medicine, stomatology, pharmacy, physiotherapy, nursing, paramedics, dietetics etc.)?

_____ (yes/no)

6. Year of studies

_____ (1-6)

Part 2.- drug usage and mental health questions

1. How do you assess in -5/0/+5 scale impact of COVID-19 pandemic situation on your tendency to:

- Drink coffee, tea, energetic drinks, sweetened caffeinated beverages or usage of caffeine supplements?
- Smoke tobacco or use other heated tobacco products (IQOS, glo or hookahs)?
- Drink alcohol?
- Use marijuana (THC) or hashish?
- Use hard drugs (heroin, cocaine, methamphetamine)?

  (Answers from -5 to -1 means negative impact, 0 means no impact and answers from +1 to +5 means positive impact)

2. How many cigarettes did you normally smoke per day during pandemic?

- 0
- <10
- 10-19
- 20-30

3. How often did you normally use other heated tobacco products* during pandemic?

- Never
- Several times per year (occasionally)
- Less than 20 days within 30 days
- 20 days within 30 days or more often
- Up to an hour very day
- More than an hour a day

*Heated tobacco products: IQOS, glo or hookahs.

4. How often did you drink one serving portion of alcohol* during pandemic?

- Never (non-drinkers)
- Several times per year (occasionally)
- Once a month
- Once a week
- 3-4 times a week
- Every day

* One serving portion (10g of ethanol) = 250ml of 5% beer, 30 ml of 40% vodka or 100ml of 12% wine.

5. How do you assess in -5/0/+5 scale impact of COVID-19 pandemic situation on your mental well-being? (Answers from -5 to -1 means negative impact, 0 means no impact and answers from +1 to +5 means positive impact)

POLISH Version
Część 1.- pytania ogólne i demograficzne

1. Płeć

   _____ (mężczyzna, kobieta, inna, wolę nie podawać)

2. Wzrost

   _____ (w cm)

3. Waga

   _____ (kg)

4. Wiek

   _____ (lata)

5. Czy jesteś studentką/studentem kierunku medycznego? (Kierunek lekarski, lekarsko-dentystyczny, farmacja, fizjoterapia, pielęgniarstwo, ratownictwo medyczne, dietetyka itp.)?

   _____ (tak/nie)

6. Rok studiów

   _____ (1-6)

Część 2.- pytania dotyczące stosowania używek i zdrowia psychicznego

1. Jak oceniasz wpływ pandemii COVID-19 na tendencję do korzystania przez Ciebie z podanych poniżej używek:

- Spożycie kawy, herbaty, napojów energetyzujących, napojów typu cola lub suplementów zawierających kofeinę?
- Palenie tytoniu lub stosowanie innych wyrobów zawierających nikotynę (IQOS, glo lub fajki wodnej)?
- Spożycie alkoholu?
- Przyjmowanie marihuany (THC) lub haszyszu?
- Przyjmowanie tzw. twardych narkotyków (heroina, kokaina, metamfetamina)?

(Wartości od -5 do -1 oznaczają zmniejszenie tendencji, a od +1 do +5 zwiększenie; 0 oznacza brak wpływu)

2. Średnio jak dużo papierosów paliłaś/eś dziennie w trakcie pandemii?

- 0
- <10

- 10-19
- 20-30

3. Średnio jak często korzystałaś/eś z e-papierosów i/lub podgrzewanych wyrobów tytoniowych, tj. IQOS, glo lub fajki wodnej w trakcie pandemii?

- Nigdy
- Kilka razy w ciągu roku (okazjonalnie)
- Rzadziej niż 20 dni w ciągu 30 dni
- 20 dni w ciągu 30 dni lub częściej
- Codziennie łącznie do godziny
- Codziennie łącznie ponad godzinę

4. Jak często spożywałaś/eś porcję napoju alkoholowego* w trakcie pandemii?

- Nie piję alkoholu
- Kilka razy w roku (okazjonalnie)
- Raz w miesiącu
- Raz w tygodniu
- 3-4 razy w tygodniu
- Codziennie

* Porcja napoju alkoholowego (równowartość 10g etanolu) = 250ml 5% piwa, 30 ml 40% wódki lub 100ml 12% wina.

5. Jak ocenisz wpływ pandemii COVID-19 na Twoje zdrowie psychiczne oraz samopoczucie? (Wartości od -5 do -1 oznaczają zmniejszenie tendencji, a od +1 do +5 zwiększenie; 0 oznacza brak wpływu)
